# TRPV1^+^ neurons alter *Staphylococcus aureus* skin infection outcomes by affecting macrophage polarization and neutrophil recruitment

**DOI:** 10.1186/s12865-023-00584-x

**Published:** 2023-12-21

**Authors:** Changyu Huang, Yang Chen, Yuanqing Cai, Haiqi Ding, Jiaoying Hong, Shan You, Yiming Lin, Hongxin Hu, Yongfa Chen, Xueni Hu, Yanshu Chen, Ying Huang, Chaofan Zhang, Yunzhi Lin, Zida Huang, Wenbo Li, Wenming Zhang, Xinyu Fang

**Affiliations:** 1 Department of Orthopaedic Surgery, National Regional Medical Center, Binhai Campus of the First Affiliated Hospital, Fujian Medical University, Fuzhou, 350212 China; 2 Department of Orthopedic Surgery, The First Affiliated Hospital of Fujian Medical University, Fuzhou, 350000 China; 3 Fujian Provincial Institute of Orthopedics, the First Affiliated Hospital, Fujian Medical University, Fuzhou, 350000 China; 4 Department of Orthopaedics, The Second Affiliated Hospital of Xi’an Jiaotong University, Xi’an, Shaanxi China; 5 Department of Anesthesiology, The Second Hospital of Nan’an, Quanzhou, Fujian China; 6 Fujian Medical University, Fuzhou, Fujian China; 7 Department of Laboratory Medicine, The First Affiliated Hospital of Fujian Medical University, Fuzhou, China; 8 Department of Stomatology, the First Affiliated Hospital, Fujian Medical University, Fuzhou, China; 9 Fuzhou, China

**Keywords:** Staphylococcus aureus, Nociceptor neuron, Calcitonin gene-related peptide, Macrophage, Neutrophile, Skin infection

## Abstract

**Background:**

The interaction between the nervous system and the immune system can affect the outcome of a bacterial infection. *Staphylococcus aureus* skin infection is a common infectious disease, and elucidating the relationship between the nervous system and immune system may help to improve treatment strategies.

**Results:**

In this study, we found that the local release of calcitonin gene-related peptide (CGRP) increased during *S. aureus* skin infection, and *S. aureus* could promote the release of CGRP from transient receptor potential cation channel subfamily V member 1 (TRPV1^+^) neurons in vitro. The existence of TRPV1^+^ neurons inhibited the recruitment of neutrophils to the infected region and regulated the polarization of macrophages toward M2 while inhibiting polarization toward M1. This reduces the level of inflammation in the infected area, which aggravates the local infection. Furthermore, this study demonstrates that TRPV1 may be a target for the treatment of *S. aureus* skin infections and that botulinum neurotoxin A (BoNT/A) and BIBN4096 may reverse the inhibited inflammatory effect of CGRP, making them potential therapeutics for the treatment of skin infection in *S. aureus*.

**Conclusions:**

In *S. aureus* skin infection, TRPV1^+^ neurons inhibit neutrophil recruitment and regulate macrophage polarization by releasing CGRP. BoNT/A and BIBN4096 may be potential therapeutic agents for *S. aureus* skin infection.

## Introduction

Skin is an important barrier for resistance to invasion by pathogenic microorganisms and direct contact with the external environment. When the integrity of the skin is compromised or the immune function of the body is disturbed, microorganisms can invade the epidermis, dermis, and subcutaneous tissues, causing skin and soft tissue infections (SSTI) [1]. SSTI is one of the most common infections in all age groups [2] and is mainly caused by septic bacteria such as *Staphylococcus* and *Streptococcus* but also by viruses, mycobacteria, and fungi [3, 4], with *Staphylococcus aureus* considered the most common causative agent [5]. Recent studies have shown that immune cells in the skin are in close contact with nociceptor neurons and express neuropeptides and neurotransmitter receptors, thus enabling immune cells to participate in neural modulation and respond rapidly to injurious stimuli [6].

TRPV1^+^ (transient receptor potential cation channel subfamily V member 1) neurons are an important subset of nociceptor neurons that are widely distributed in the skin [6]. They sense various types of injurious stimuli, elicit action potentials, and conduct information from injury stimuli from the skin to the dorsal root ganglia (DRG) and central nervous system to cause nociception [6]. TRPV1 is a nonselective cation channel that can be activated by heat (> 43 °C), acids, lipids, and vanilloids (e.g., capsaicin) [7]. Recent studies have found that in addition to cytokines released during the inflammatory response (e.g., IL-1β and TNF-α) that can activate nociceptor neurons expressing the corresponding receptors, some components of pathogenic bacteria can also directly activate nociceptor neurons [8, 9]. Formyl peptides from heat-killed *S. aureus* and HLα (a pore-forming toxin) from *S. aureus* can directly activate nociceptor neurons to cause pain [10]. Moreover, Streptolysin S (a pore-forming toxin) of *Streptococcus pyogenes* origin causes pain as well as morbidity by inducing calcium influx in TRPV1^+^ neurons [11]. β-Glucan secreted by *Candida albicans* causes nociception through Dectin1-PLC-TRPV1/TRPA1 axis activation of nociceptor neurons [12]. Additionally, lipopolysaccharide from the cell wall of gram-negative bacteria binds to Toll-like receptors (TLR4) and CD14 on TRPV1^+^ neurons to induce pain [13].

In response to bacterial stimulation, nociceptor neurons release neuropeptides and neurotransmitters such as calcitonin gene-related peptide (CGRP) to regulate immune responses. CGRP is a long peptide of 37 amino acids produced by sensory nerves or immune cells [14], which regulates blood pressure and potentially relieves migraines [15]. CGRP is primarily associated with type-I transmembrane protein receptor activity modifying protein-1 (RAMP1) and calcitonin receptor-like receptor (CLR) binding to produce biological effects [16]. Many immune cells, including macrophages and dendritic cells, among others, have been found to express RAMP1 and CLR [14, 17]. Additionally, macrophages are important regulators in infectious inflammation, as they can change their phenotype and thus change their function depending on the local environment. These phenotypes are summarized as follows: classical activated macrophages (M1) induced by LPS and IFN-γ secrete proinflammatory factors such as IL-1β, IL-6, and TNFα and express CD80/CD86; M2 macrophages induced by IL-4 and IL-13, secrete anti-inflammatory factors such as IL-10 and TGFβ and express CD163/CD206 [18]. Macrophages regulate local tissue homeostasis by switching between these two phenotypes (proinflammatory or anti-inflammatory) [19], and they play an important role in SSTI as important immune cells in the skin. Currently, the crosstalk between TRPV1^+^ neurons and macrophages in SSTI has not been fully elucidated.

In this study, we found that selective knockout or ablation of TRPV1 improved the outcome of SSTI with *S. aureus* and reduced the rate of skin breakdown in mice. *S. aureus* activates TRPV1^+^ neurons in the skin and leads to the release of CGRP. We found that the existence of TRPV1^+^ neurons not only inhibited the recruitment of neutrophils to the infected area, but also promoted the conversion of macrophages in the infected region to the M2 phenotype, which led to a decrease in the clearance of *S. aureus* from the animal. Furthermore, after we antagonized the effect of CGRP with BIBN4096 or blocked the release of CGRP with botulinum neurotoxin A (BoNT/A), the skin infection outcome of mice was improved, indicating that BIBN4096 and BoNT/A may be potentially effective therapeutic agents for SSTI.

## Materials and methods

### Animals

All animal care protocols and experiments were approved by the Animal Research Committee of our institution (IACUC FJMU 2022-NSFC-0368) and were performed according to international standards. C57BL/6 mice (wild-type, WT) were purchased from Beijing HFK Bioscience Co., Ltd., and TRPV1^−/−^ mice (Cat. NO. NM-KO-210259, NCBI ID: 193034) were purchased from Shanghai Model Organisms Center, Inc.. All animals were housed in individually ventilated microisolation cages with free access to water and food, a temperature of 22–24 °C, a humidity of 60 ± 5%, and a 12-h light/dark cycle. Age-matched healthy female or male mice of 4–14 weeks of age were used for this study. The animals were euthanized by CO_2_ inhalation.

### Bacterial culture and infection

This study was performed using the standard strain of *Staphylococcus aureus* (USA300). All operations involving *S. aureus* were performed according to Biosafety Level 2 protocols and guidelines. *S. aureus* was stored at -80 °C using the glycerol freezing method. Bacterial fluids were quantified by optical density before use by resuscitation at 37 °C in 5% CO_2_ for 18–22 h. Prior to modeling, mice were anesthetized with precision vaporizer (2–3% isoflurane), the dorsal skin was shaved with a razor, and hair removal was performed with hair removal cream (Nair). *S. aureus* was resuspended in 0.1 ml PBS according to experimental needs and injected subcutaneously with a 1 ml syringe. Mice were kept in a warm environment until active and then were moved into isolation cages. The body weight, core body temperature, and skin condition of the mice were monitored daily before and after the injection until the observed endpoint.

### Bacterial load recovery analysis

After euthanasia of the mice, the tissues (including epidermis, dermis, and subcutaneous tissue) of the infected area of the back were quickly removed, weighed, maintained at 4 °C, and transferred to the microbiology lab. Then, tissues were placed in sterile test tubes, 1 mL of brain heart infusion broth was added, and the samples were vortexed and shaken for 15 min after sealing and placed in a fully automatic rapid grinding instrument (Jingxin Industrial Development, Shanghai, China) at 60 Hz for 60 s until the tissue was homogenized. The obtained homogenate was diluted in a gradient and inoculated on a blood agar plate (Thermo Fisher Scientific, USA).

### Resiniferatoxin, BoNT/A and BIBN4096 treatments

Resiniferatoxin (RTX) can destroy TRPV1^+^ neurons [20]. To destroy TRPV1^+^ neurons with RTX (Sigma‒Aldrich, #R8756), 4-week-old WT mice were anesthetized and injected subcutaneously with RTX (30 μg/kg, 70 μg/kg, 100 μg/kg) or vehicle (PBS with 1.2% DMSO and 0.06% Tween 80) on the backs of the mice for three consecutive days. Experiments were performed on the mice 4 weeks later. For the BoNT/A experiments, the dorsal skin area chosen for infection was injected 7 days before infection with BoNT/A (25 pg in 0.1 ml PBS) or vehicle (PBS). For the BIBN4096 experiments, one intraperitoneal injection of BIBN4096 (30 mg/kg, Tocris, #4561) or vehicle (PBS) was administered 2 h postinfection, followed by one injection daily until the observed endpoint.

### Isolation and culture of DRG neurons

Immediately after euthanasia of 6-week-old mice, DRG neurons were isolated from each segment of the spinal cord. The cells were washed, transferred into 2 ml of collagenase D (Roche, #11088858001) solution (0.7 mg/ml), counted, and placed in a 37 °C, 5% CO_2_ incubator for 30 min. After the supernatant was collected, 2 ml of collagenase D was replaced and incubated again for 30 min, and the supernatant was collected a second time. 1 ml of DMEM/F12 (Meiluncell®, #MA0214) containing 10% FBS and 150 U DNAase I (Thermo Fisher, #18047019) was added. The cell suspension was mixed 60 times. The cell suspension was then filtered with 70 μm mesh, washed and resuspended in new cell culture medium, and then inoculated in rat tail tendon collagen type I (2 µg/cm^2^, Meiluncell®, #MB5680) precoated culture dishes. Cell culture medium for the first 48 h was as follows: DMEM/F12, 10% FBS, 1% NEAA (Meiluncell®, #PWL088), NGF (50 ng/ml, Novoprotein, #CK21), and kanamycin (100 mg/ml, Meiluncell®, #MA0134). After 48 h, the medium was changed to DMEM/F12, 1% NEAA, NGF (50 ng/ml), and kanamycin (100 mg/ml). Approximately 5000 cells per culture dish were used for the CGRP release experiments.

### Isolation and culture of bone marrow-derived macrophages

Macrophages were isolated from mice femoral bone marrow according to a previously reported method [21]. Primary macrophages were treated with macrophage colony-stimulating factor (20 ng/ml, Novoprotein, #CK02) in RPMI-1640 (Meiluncell®, #MA0215) culture medium for 7 days. Then, after treatment with CGRP (100 nM, GenScript, #RP11095) or vehicle for 2 h, M1 polarization was induced with IFN-γ (10 ng/ml, Novoprotein, #CM41), M2 polarization was induced with IL-4 (40 ng/ml, Novoprotein, #CK74), and subsequent experiments were performed 24 h later.

### Histology

The removed tissues were placed in 4% paraformaldehyde for 3 days. After dehydration, the tissues were embedded in paraffin blocks and cut into 5-μm coronal slides by a microtome. Then, the cells were stained with hematoxylin and eosin (H&E) (Beyotime, #C0105S).

### Immunofluorescence staining

The isolated tissue was cut into 5-μm sections. The tissue sections were treated in PBS containing 1% BSA (Meiluncell®, #MB4219) and 0.3% Triton for 1 h at room temperature. Then, they were incubated overnight at 4 °C with the following primary antibodies (1:200): anti-TRPV1 (Abcam, #ab3487), anti-CGRP (Abcam, #ab18207), and anti-TUBB3 (Abcam, #ab18207). After washing the primary antibody, they were incubated with Alexa 594 (Abcam, 1:500) and DyLight 488 (Abcam, 1:500) for 1 h at room temperature to fluorescently label the target proteins. For cytological immunofluorescence, cells were isolated and fixed with 4% PFA for 10 min and then treated with 1% BSA and 0.1% Triton in PBS for 30 min. The remaining steps were the same as those used for histological immunofluorescence staining. Randomly selected fluorescence images were obtained with a Zeiss microscope (Zeiss, Germany).

### Flow cytometry

The removed tissue from the infected area was added to 2 ml of HEPES buffer containing collagenase A (1 mg/kg, Roche, #10103586001) and Dispase II (2.4 U/ml, Sigma, #D4693), and then the tissue was incised and placed in a water bath at 37 °C for 2 h. The cell suspension was obtained by diluting the tissue pieces with PBS buffer containing 5% FBS and filtering it through a 70 μm mesh. After washing, the cell suspension was subjected to gradient centrifugation (25 °C, 750 g, 20 min) with 40% and 80% Percoll (Cytiva, #17089101) to extract the white film layer. Single-cell suspensions were made by resuspension in buffer after washing. The single-cell suspension was incubated with an FcR blocker (Elabscience) on ice for 10 min. Then, the suspension was incubated on ice for 30 min with the following antibodies (1:200): anti-CD11b (Invitrogen, #12–0112-82), anti-F4/80 (Invitrogen, #17-4801-82), and anti-CD80 (Elabscience, #E-AB-F1135D). After washing, the cells were fixed/permeabilized according to the instructions provided by the manufacturer (MultiSciences, FoxP3/Transcription Factor Staining Buffer Kit) and then incubated on ice with CD206 (Elabscience, #E-AB-F0992C) for 30 min. Flow cytometry data were collected and exported using Beckman CytoFLEX (USA). FACS data were analyzed and plotted using FlowJo software (10.8.1, USA).

### Immunohistochemical staining

Following paraffin dehydration and clearing and antigen retrieval, endogenous peroxidase activity was quenched via incubation with 3% H_2_O_2_ for 15 min. Primary antibodies (1:200) for anti-CD80 (Sigma, #MABF455), anti-CD206 (Sigma, #AMAB90746) and anti-F4/80 (Sigma, #SAB5500103) were used, followed by incubation with a horseradish peroxidase-conjugated goat anti-rabbit immunoglobulin G (1:1000, Sigma, #SAB4200801) secondary antibody. Immunohistochemical staining was visualized using an ultrasensitive DAB kit (Servicebio, #G1211). For image analysis, the area with the highest concentration of cells near the infection area was selected under the low magnification field, and then a visual field was randomly intercepted for analysis under the high magnification field. ImageJ (National Institutes of Health, V1.8.0) was used to analyze the acquired images.

### Enzyme-linked immunosorbent assay (ELISA)

ELISA kits for CGRP (Sigma, #C7113), IL-1β (MultiSciences, #70-EK201BHS-96), TNF-α (MultiSciences, #70-EK282HS/3–96), and IL-10 (MultiSciences, #70-EK210/4–96) were used to detect the levels of each protein in the cell culture supernatant according to the manufacturer’s instructions. For tissues, the removed specimens were placed in a 24-well plate containing 1 ml of DMEM and placed on a shaker at 32 °C (150 rpm) for 45 min, and then the supernatant was collected and later analyzed for the levels of each protein by the ELISA kits.

### In vivo bioluminescence imaging of myeloperoxidase (MPO)

Bioluminescence imaging was performed with an IVIS Lumina X5 System (PerkinElmer, USA). Mice were lightly anesthetized with isoflurane. Luminol (25 mg/kg, Sigma, #123072) was injected intraperitoneally into the mice for 5 min, and imaging was started [22]. Isoflurane anesthesia was maintained over the course of imaging. Bioluminescence data were acquired and analyzed with Living Image software (V4.7.4.21053, PerkinElmer, USA).

### Determination of MPO activity

The activity of MPO was determined using a kit (Sigma, #MAK068) according to the manufacturer’s instructions. MPO activity was measured spectrophotometrically at 412 nm absorbance. MPO activity was defined as the quantity of enzyme degrading 1 mmol of peroxide per min at 37 °C.

### Statistical analysis

Statistical analysis was performed using SPSS (V26.0, IBM, USA) and GraphPad Prism (V9.0.0, GraphPad Software). Data are expressed as the mean ± SEM. The t test and one-way ANOVA were used for comparisons between groups. *P* < 0.05 was considered statistically significant. Data were pooled from two or three independent experiments. Statistical significance was assessed as follows: *p < 0.05, **p < 0.01, ***p < 0.001, ****p < 0.0001, ns, not significant.

## Results

### Knockout or ablation of TRPV1+ neurons can reduce the *S. aureus* burden in the infected area and reduces the rate of skin breakdown.

**Fig. 1:**
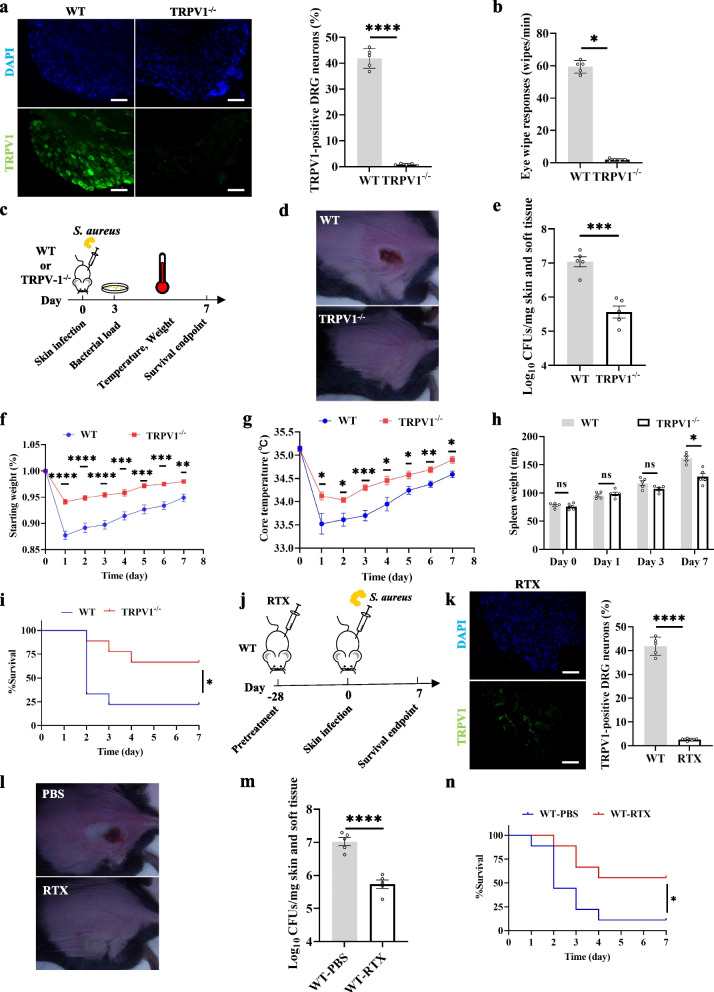
Knockout or ablation of TRPV1^+^ neurons can reduce the *S. aureus* burden in the infected area and reduces the rate of skin breakdown. **a** Isolated DRG from WT and TRPV1^−/−^ mice were stained with DAPI (blue), TRPV1 (green); the proportion of TRPV1-positive regions in DRG of different groups of mice was analyzed (*n* = 3/group); Scale bars, 100 μm; unpaired *t* test. **b** Number of spontaneous eye wipes per minute in TRPV1^−/−^ or WT mice after eye drops with capsaicin (*n* = 5/group); unpaired *t* test. **c** The skin of TRPV1^−/−^ or WT mice was infected with 1 × 10^8^ CFUs of *S. aureus* and subsequently tested for various indices. **d** Photograph of TRPV1^−/−^ and WT mice after 3 days of skin infection. **e** Bacterial load in skin tissue in TRPV1^−/−^ and WT mice after 3 days of infection (*n* = 5/group); unpaired* t* test. **f** Comparison of weight in TRPV1^−/−^ and WT mice after infection (*n* = 9/group); multiple unpaired *t* test. **g** Comparison of core body temperature in TRPV1^−/−^ and WT mice after infection (*n* = 9/group); multiple unpaired *t* test. **h** Comparison of spleen weight in TRPV1^−/−^ and WT mice after infection (*n* = 5/group); multiple unpaired *t* test. **i** Skin breakout survival curves for TRPV1^−/−^ or WT mice (*n* = 9/group); log-rank (Mantel‒Cox) test. **j** Pretreatment of 4-week-old WT mice with RTX or PBS and skin infection 28 days later, followed by analysis of the indices. **k** Isolated DRG from WT and RTX-pretreated mice were stained with DAPI (blue), TRPV1 (green); the proportion of TRPV1-positive regions in DRG of different groups of mice was analyzed (*n* = 3/group); Scale bars, 100 μm; unpaired *t* test. **l** Typical photograph of the RTX and PBS groups after 3 days of skin infection. **m** Bacterial load in skin tissue after 3 days of infection in the RTX and PBS groups (*n* = 5/group); unpaired *t* test. **n** Skin breakdown survival curves in the RTX and PBS groups (*n* = 11/group); log-rank (Mantel‒Cox) test. Data were pooled from two or three independent experiments

We used mice with TRPV1 channels selectively knocked out (TRPV1^−/−^) for skin infection modeling. Immunofluorescence of the DRG suggested a significant reduction of TRPV1^+^ neurons (Fig. 1a). Capsaicin is an agonist of TRPV1, and a capsaicin swabbing test performed prior to modeling confirmed the absence of TRPV1 function (Fig. 1b) [23]. The infection model was initiated by subcutaneously injecting 1 × 10^8^ CFUs of *S. aureus* into the backs of TRPV1^−/−^ and C57BL/6 (wild-type, WT) mice (Fig. 1c). After 3 days of infection, the skin bacterial load of TRPV1^−/−^ mice was significantly decreased compared to that of WT mice (Fig. 1d, e). Moreover, TRPV1^−/−^ mice showed a significant improvement in body weight maintenance (Fig. 1f) as well as core body temperature maintenance (Fig. 1g) during infection compared to those of WT mice. At the observed endpoint, the spleen weights of mice were compared after euthanasia, and there was no significant difference in spleen weight between the two groups on days 1 and 3; however, on day 7, TRPV1^−/−^ mice had significantly lower spleen weights than WT mice (Fig. 1h). Skin breakdown survival curves plotted after 7 days of observation suggested a significant decrease in the skin breakdown rate in TRPV1^−/−^ mice compared to that of WT mice (Fig. 1i).

Resiniferatoxin (RTX) is a potent capsaicin that lesions TRPV1^+^ neurons [24]. We lesioned TRPV1^+^ neurons with RTX 4 weeks prior to infection (Fig. 1j), and DRG immunofluorescence suggested a significant reduction in TRPV1^+^ neurons (Fig. 1k). Three days after infection, the skin bacterial load of mice pretreated with RTX was significantly reduced compared to that of the PBS group (Fig. 1l, m). Survival curves suggested a significant decrease in the skin breakdown rate in the RTX group (Fig. 1n). Skin infection outcomes improved in whether TRPV1^+^ neurons were knocked out or eliminated in mice. This finding suggests that TRPV1^+^ neurons inhibit the mice skin defense response against *S. aureus* during infection.

### TRPV1^+^ neurons inhibit the recruitment of neutrophils to the infected area

**Fig. 2:**
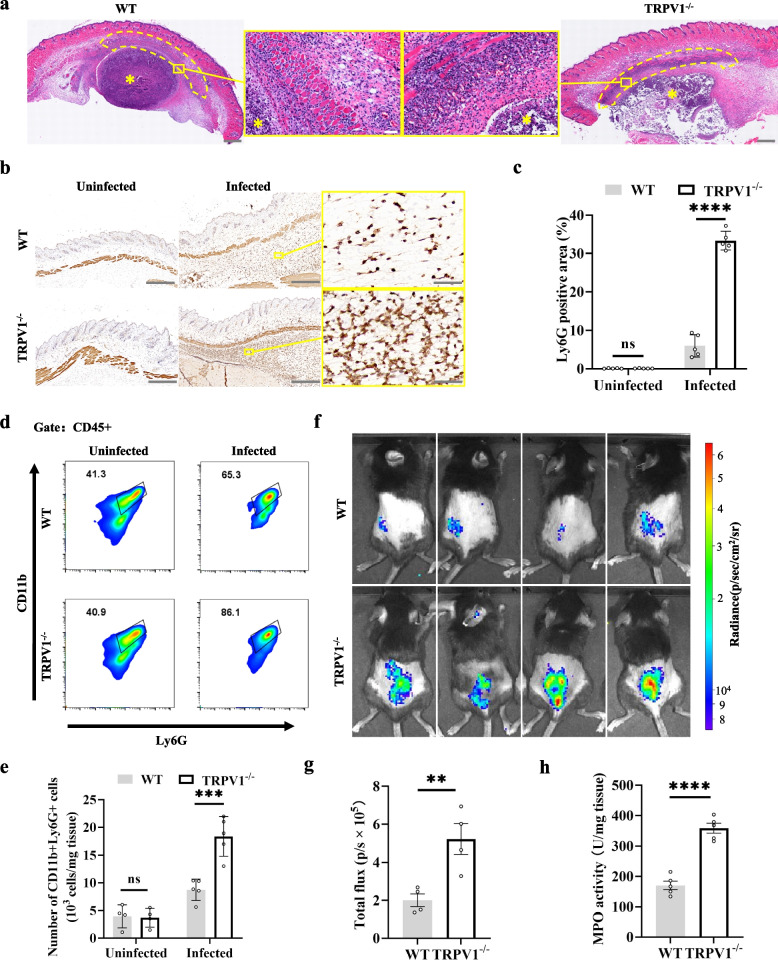
TRPV1^+^ neurons inhibit the recruitment of neutrophils to the infected area. **a** H&E staining of tissue after 2 days of infection, with more nucleated cell recruitment seen in the TRPV1^−/−^ infected area; yellow border shows high magnification images; difference in nucleated cell recruitment seen in the dashed area; Scale bars, 500 μm; High magnification images, scale bars, 50 μm; *abscess. **b**, **c** IHC staining of TRPV1^−/−^ and WT mouse skin and analysis of the proportion of Ly6G-positive areas; scale bars, 500 μm; high magnification images, scale bars, 50 μm; unpaired *t* test. **d**, **e** Flow cytometric analysis of skin neutrophil ratios in TRPV1^−/−^ and WT mice (*n* = 5/group); unpaired *t* test. **f**, **g** Bioluminescence in vivo imaging of MPO in TRPV1^−/−^ and WT mice (*n* = 4/group); unpaired *t* test. **h** Comparison of MPO levels in TRPV1^−/−^ and WT mice (*n* = 5/group); unpaired *t* test. Data were pooled from two or three independent experiments

Next, we asked what effect TRPV1^+^ neurons have on the immunity of the infected skin area. In order to avoid the confounding factors caused by skin breakdown, including in vivo bioluminescence imaging distortion and wound infection with other pathogenic bacteria, we used a dose of 1 × 10^7^ CFUs of S. aureus to reduce the rate of skin breakdown. The skin of TRPV1^−/−^ and WT mice was infected with 1 × 10^7^ CFUs of *S. aureus*. Two days later, H&E staining of tissue sections suggested a significant increase in the number of nucleated cells recruited subcutaneously in TRPV1^−/−^ mice compared to WT mice (Fig. 2a). Neutrophils respond rapidly and are recruited in large numbers to an infected area to attack microorganisms, so we first asked whether the number of neutrophils differed between groups. Immunohistochemistry of tissue sections 1 day after infection suggested that TRPV1^−/−^ mice had significantly more Ly6G + cells in the infected region than those in the infected region of WT mice (Fig. 2b, c). Similarly, flow cytometry results 1 day after infection suggested a higher number of subcutaneously recruited neutrophils in TRPV1^−/−^ mice than that in WT mice (Fig. 2d, e). Myeloperoxidase (MPO), a peroxidase that is abundantly expressed in neutrophils and macrophages, has antimicrobial effects [25]. We labeled MPO with luminol, and the bioluminescence results showed significantly higher local MPO activity in TRPV1^−/−^ mice than that in WT mice 1 day after infection (Fig. 2f, g). Likewise, tissue MPO activity assays suggested significantly higher MPO expression in TRPV1^−/−^ mice than in that in WT mice (Fig. 2h). The above data suggest that TRPV1^+^ neurons inhibit the recruitment of neutrophils to the infected region during *S. aureus* infection and lead to a decrease in MPO expression, resulting in a decrease in the ability of the animal to resist *S. aureus*.

### TRPV1^+^ neurons affect the polarization of local macrophages in the skin

**Fig. 3:**
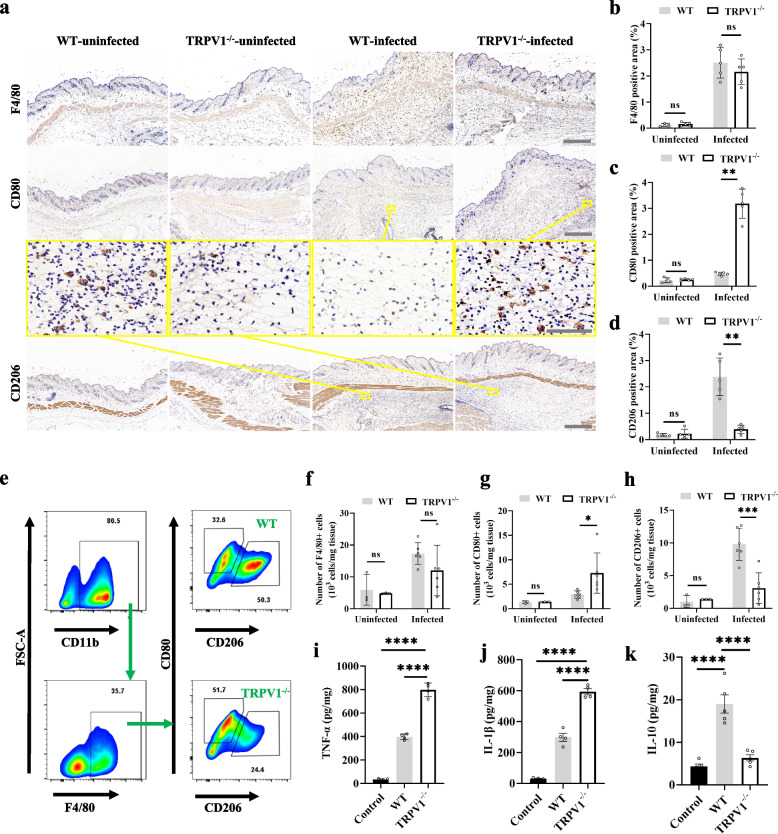
TRPV1^+^ neurons affect the polarization of local macrophages in the skin. **a** IHC staining of the skin of TRPV1^−/−^ and WT mice; scale bars, 500 μm; high magnification images, scale bars, 50 μm. **b**-**d** IHC analysis of the ratio of M0 (F4/80 +), M1 (CD80 +) and M2 (CD206 +) cells in TRPV1^−/−^ and WT mice (*n* = 5/group); unpaired t test. **e**–**h** Flow cytometric analysis of M0 (CD11b + F4/80 +), M1 (CD11b + F4/80 + CD80 +), and M2 (CD11b + F4/80 + CD206 +) ratios in the skin of TRPV1^−/−^ and WT mice (*n* = 5/group); unpaired *t* test. **i**-**k** Tissue expression levels of TNFα, IL-1β and IL-10 in TRPV1^−/−^ and WT mice 1 day after skin infection; the control group was uninfected WT mice; One-way ANOVA with Tukey’s post hoc test. Data were pooled from two or three independent experiments

Macrophages are important immune cells in skin tissues; here, we asked whether TRPV1^+^ neurons affect macrophages in infected areas. We performed immunohistochemical staining of isolated skin tissues to explore local macrophage phenotypic changes. After 1 day of infection, the total number of macrophages (M0, F480 +) in the infected areas of TRPV1^−/−^ and WT cells did not differ significantly (Fig. 3a, b). However, the number of M1 (CD80 +) cells was significantly increased in TRPV1^−/−^ cells compared to that in WT cells (Fig. 3a, c), while the number of M2 (CD206) cells was significantly decreased compared to that in WT cells (Fig. 3a, d). Since dendritic cells also upregulate the expression of CD80 during inflammation, we further detect the phenotypic changes of local macrophages by flow cytometry. After infection, there was no significant difference in the number of M0 (F4/80 +) cells between TRPV1^−/−^ and WT cells (Fig. 3f); however, the number of M1 (F4/80 + CD80 +) cells was significantly higher in TRPV1^−/−^ cells than that in WT cells (Fig. 3e, g), whereas the number of M2 (F4/80 + CD206 +) cells was lower than that in WT cells (Fig. 3e, h). M1 mainly secretes IL-1β, TNFα and other proinflammatory factors to promote local inflammatory responses, while M2 secretes IL-10 and other cytokines to suppress inflammation [26]. Additionally, ELISA results of the postinfection skin samples suggested that the expression of IL-1β and TNFα increased in TRPV1^−/−^ mice compared to those in WT mice (Fig. 3i, j), while the expression level of IL-10 decreased compared to that in WT mice (Fig. 3k). The above results indicate that although TRPV1^+^ neurons have no significant effect on macrophage recruitment in the infected area of the skin, they regulate macrophage polarization, which inhibits M1 polarization and promotes M2 polarization. This change in macrophage polarization may down-regulate the release of pro-inflammatory factors, which is not conducive to the clearance of *S. aureus*.

### *S. aureus* activates cutaneous TRPV1^+^ neurons to release CGRP

**Fig. 4:**
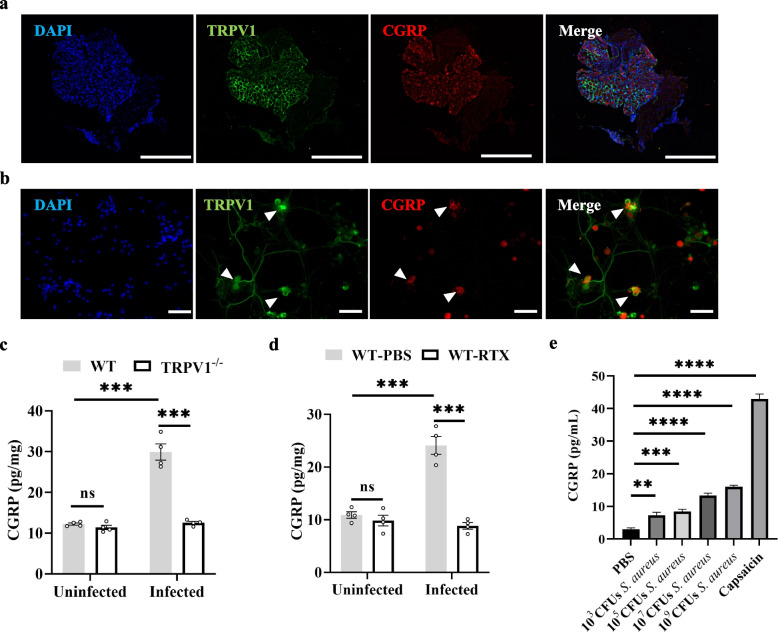
*S. aureus* activates cutaneous TRPV1^+^ neurons to release CGRP. **a** DRG was isolated from WT mice and stained with DAPI (blue), TRPV1 (green), and CGRP (red); scale bars, 200 μm. **b** Primary DRG neurons isolated from 6-week-old WT mice were stained with DAPI (blue), TRPV1 (green), and CGRP (red); white arrows show colocalization of TRPV1 with CGRP; scale bars, 50 μm. **c** CGRP release from dorsal skin tissue of TRPV1^−/−^ and WT mice before and 8 h after infection (*n* = 4/group); One-way ANOVA with Tukey’s post hoc test. **d** Pretreatment of mice with RTX or PBS and CGRP release from dorsal skin tissue before and 8 h after infection (*n* = 4/group); One-way ANOVA with Tukey’s post hoc test. **e** DRG neurons were stimulated with different concentrations of *S. aureus* supernatant or capsaicin for 30 min, and the release of CGRP was analyzed (*n* = 4/group). One-way ANOVA with Tukey’s post hoc test. Data were pooled from two or three independent experiments

Previous studies have reported that *S. aureus* stimulates TRPV1^+^ neurons in the lung, leading to a significant increase in CGRP secretion [27]. Here, we hypothesized that TRPV1^+^ neurons increase the release of CGRP during *S. aureus* skin infection. Sensory nerve fibers from the DRG innervate most of the skin, and TRPV1^+^ neurons are among these sensory nerves [28]. Immunofluorescence staining of the DRG showed that TRPV1 was expressed in the DRG neurons and colocalized with CGRP (Fig. 4a). We isolated and extracted primary neurons from the DRG for in vitro culture, and immunofluorescence suggested the presence of TRPV1^+^ neurons, and the proximity of TRPV1^+^ neurons to CGRP was observed (Fig. 4b). Skin tissues from uninfected TRPV1^−/−^ and WT mice were taken for analysis of CGRP levels, and ELISA showed no difference in the amount of CGRP between the two groups. However, assays at 8 h postinfection showed that CGRP release was significantly higher in WT mice than that in TRPV1^−/−^ mice (Fig. 4c). Pretreatment of WT mice with RTX resulted in significantly lower CGRP release after infection than that in the PBS treatment group (Fig. 4d). Next, we stimulated DRG neurons cultured in vitro with supernatants containing different concentrations of *S. aureus*, and the results showed that *S. aureus* could stimulate DRG neurons in vitro to release CGRP (Fig. 4e). These results suggest that TRPV1^+^ neurons in the infected area increase the release of CGRP during skin infection, and in vitro experiments show that the stimulation of TRPV1^+^ by *S. aureus* can increase the release of CGRP.

### CGRP regulates the polarization of bone marrow-derived macrophages (BMDMs) and the release of inflammatory factors

**Fig. 5:**
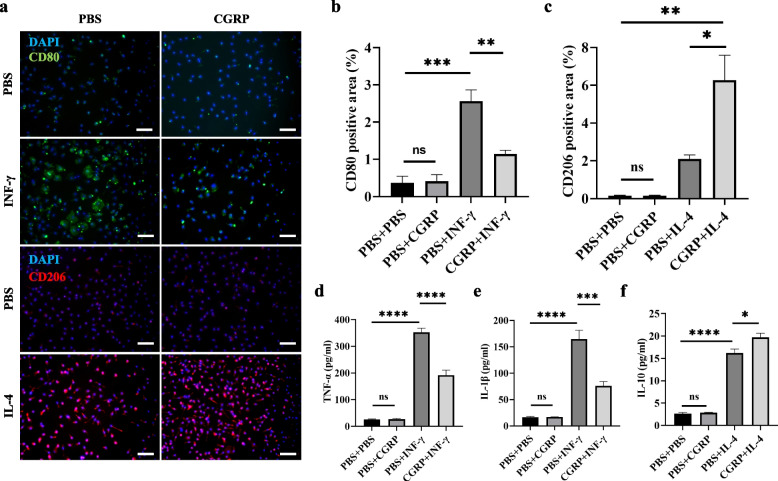
CGRP regulates the polarization of BMDMs and the release of inflammatory factors. **a**-**c** BMDMs cultured in vitro were induced to M1 polarization with IFN-γ and M2 polarization with IL-4 The polarized macrophages were treated with CGRP or PBS and stained with DAPI (blue), CD80 (green), and CD206 (red); the ratio of CD80 + and CD206 + was analyzed under different intervention conditions. No difference in the confluency of BMDMs was observed among different groups. Scale bars, 20 μm. One-way ANOVA with Tukey’s post hoc test. **d**-**f** Expression levels of TNFα, IL-1β, and IL-10 in BMDMs after polarization in vitro under different experimental conditions; one-way ANOVA with Tukey posttests. Data were pooled from two or three independent experiments

Next, we asked whether CGRP influences macrophage polarization. We extracted BMDMs from WT mice for in vitro culture. Immunofluorescence results suggested that BMDMs induced with IFN-γ showed a decreased number of CD80 + cells after pretreatment with CGRP (Fig. 5a, b). Macrophages induced with IL-4 showed an increased number of CD206 + cells after pretreatment with CGRP (Fig. 5a, c). In the absence of IFN-γ, CGRP had no significant effect on the polarization of macrophages (Fig. 5a-c). Then, we used ELISA to detect changes in inflammatory factors secreted by CGRP-treated macrophages. The results suggested that pretreatment with CGRP reduced the release of TNF-α and IL-1β from macrophages induced by IFN-γ (Fig. 5d, e). Macrophages treated with IL-4 and pretreated with CGRP showed an increased release of IL-10 (Fig. 5f). Similarly, in the absence of induced polarization, CGRP did not affect the secretion of inflammatory factors by macrophages (Fig. 5d-f). These data suggest that CGRP regulates the polarization of BMDMs, promotes macrophage polarization toward M2 and inhibits macrophage polarization toward M1, thereby mediating the secretion of the corresponding inflammatory factors.

### BoNT/A and BIBN4096 improve the outcome of *S. aureus* skin infections

**Fig. 6:**
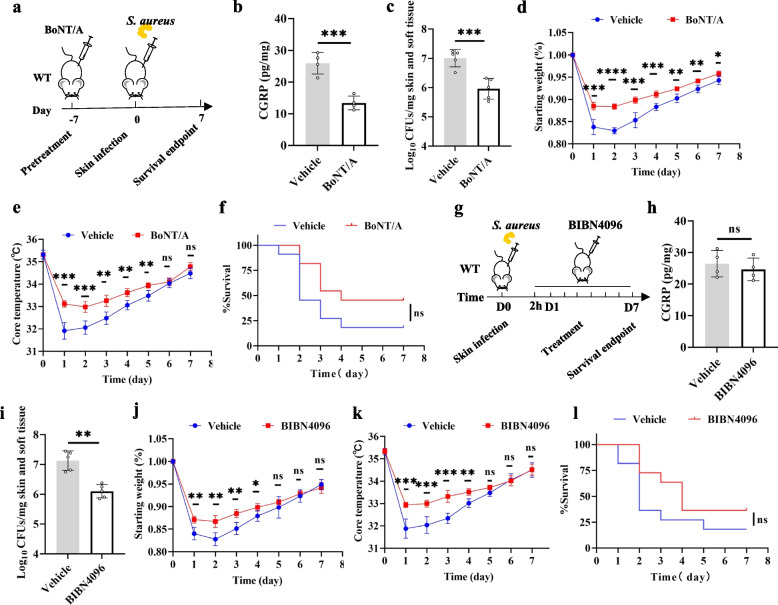
BoNT/A and BIBN4096 improve the outcome of *S. aureus* skin infections. **a** The mice were pretreated with BoNT/A or PBS 7 days before infection, and the skin was infected with 1 × 10^8^ CFUs of *S. aureus*, and the indexes were subsequently detected. **b** CGRP release from dorsal skin tissue of vehicle group and BoNT/A group 8 h after infection (*n* = 4/group); unpaired *t* test. **c** Bacterial burden of skin tissue in 2 groups of mice after 3 days of infection (*n* = 5/group); unpaired *t* test. **d**, **e** Comparison of body weight and core body temperature after infection in 2 groups of mice (*n* = 5/group); unpaired *t* test. **f** Survival curves of skin breakdown in 2 groups of mice (*n* = 11/group); log-rank (Mantel‒Cox) test. **g** The skin of WT mice was infected with 1 × 10^8^ CFUs of *S. aureus*, and the mice were treated with BIBN4096 or PBS at 2 h postinfection, and the indexes were subsequently detected. **h** CGRP release from dorsal skin tissue of vehicle group and BoNT/A group 8 h after infection (*n* = 4/group); unpaired *t* test. **i** Bacterial burden of skin tissue in 2 groups of mice after 3 days of infection (*n* = 5/group); unpaired *t* test. **j**, **k** Comparison of body weight and core body temperature after infection in 2 groups of mice (*n* = 5/group); unpaired *t* test. **l** Survival curves of skin breakdown in 2 groups of mice (*n* = 11/group); log-rank (Mantel‒Cox) test. Data were pooled from two or three independent experiments

Finally, we asked whether inhibition of CGRP could improve the outcome of mice with *S. aureus* skin infections. BoNT/A cleaves SNAP-25 in the SNARE complex, which is required for neurotransmitter release at chemical synapses, thereby blocking neuromuscular functions [29]. Mice were pretreated with BoNT/A followed by the induction of the infection model (Fig. 6a). At 8 h after infection, the level of CGRP in the infected area in the BoNT/A group was significantly lower than that in the vehicle group (Fig. 6b). After 1 day of infection, the skin *S. aureus* load of mice treated with BoNT/A was significantly reduced compared to that of the vehicle group (Fig. 6c), and the maintenance of core body temperature and body weight was improved compared to the vehicle group (Fig. 6d, e). The skin breakdown survival curves plotted after 7 days of observation suggested that although there was no significant difference between mice treated with BoNT/A compared to the vehicle group, a trend toward a decrease in breakdown rate was still seen (Fig. 6f).

BIBN4096 is a potent and selective CGRP receptor antagonist that inhibits CGRP-induced cAMP production and blocks the biological effects of CGRP [30]. WT mice were infected and treated with BIBN4096 (Fig. 6g) and compared with vehicle-treated WT mice. There was no significant difference in the level of CGRP in the infected site between the group BIBN4096 and the vehicle group at 8 h after infection (Fig. 6h). After infection, the bacterial burden, maintenance of core body temperature and body weight of BIBN4096 group were significantly better than those of vehicle group (Fig. 6i-k). The skin breakdown survival curves plotted suggested that there is a trend of improvement in BIBN4096 treatment group (Fig. 6f). These results suggest that inhibiting the release of CGRP or blocking the function of CGRP can improve the outcome of *S. aureus* skin infection. BoNT/A and BIBN4096 may be potential therapeutic drugs for *S. aureus* skin infection.

## Discussion

**Fig. 7:**
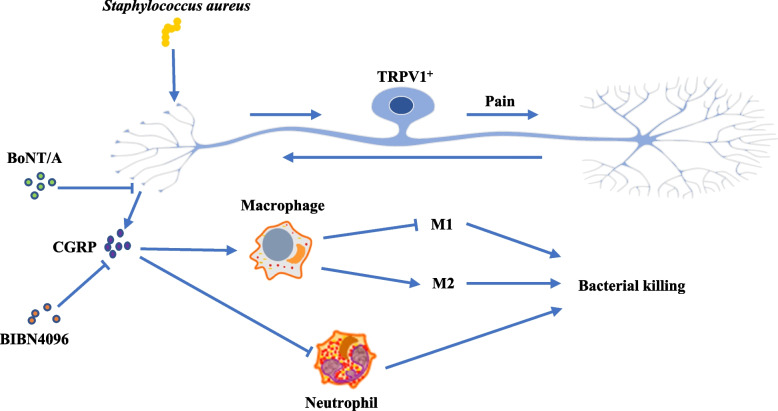
Graphical abstract. In Staphylococcus aureus skin infection, local TRPV1^+^ neurons release CGRP to inhibit neutrophil recruitment and regulate macrophage polarization, resulting in the aggravation of local infection

In this study, we demonstrated that TRPV1^+^ neurons in the infected area upregulated the release of CGRP in mice with *S. aureus* skin infection. The existence of TRPV1^+^ neurons inhibited the recruitment of neutrophils to the infected region and was involved in the regulation of macrophage polarization. In vitro, CGRP promoted macrophage polarization toward M2 while inhibiting M1 classical activation of macrophages. Moreover, the release of cytokines that promote the inflammatory response (IL-1β and TNFα) was suppressed after bacterial invasion, while the release of cytokines that suppress the inflammatory response (IL-10) was increased. The modulation of TRPV1^+^ neurons caused the immune system to decrease the clearance of invading *S. aureus*, which exacerbated the damage of *S. aureus* to the animal. When we used BoNT/A to block the vesicle release of neurons or BIBN4096 to antagonize CGRP receptors, the outcome of SSTI in *S. aureus* was improved (Fig. 7).

The nervous system maintains the dynamic homeostasis of the body through neural processes and interactions between the nervous and immune systems play an important role in the local tissue response to infection. When mice that are deficient in NAV1.8, an injury sensory neuron ion channel associated with pain, are infected with *S. aureus,* they exhibit increased lymph node size and elevated numbers of local B cells, T cells and monocytes [10]. Moreover, noradrenergic nerve fibers regulate invariant NKT cells in the liver, and blocking this innervation resulted in effective control of bacterial infection in WT mice in a stroke model [31]. Nociceptor neurons in the gut mediate host defense against *Salmonella* by regulating the abundance of Peyer’s Patch M cells and segmented filamentous bacteria [32]. In cutaneous *Candida albicans* infection, nociceptor neurons can be directly activated and promote IL-23 production by CD301b + dendritic cells, leading to IL-17A production by γδT cells and subsequent resistance to *Candida albicans* [33]. In our study, when TRPV1 was selectively knocked out, the animal improved the clearance of *S. aureus,* and infection was better controlled, suggesting that TRPV1^+^ neurons play a negative regulatory role in local immunity. This negative regulation is also present in necrotizing fasciitis caused by *Streptococci*, where nociceptor neurons respond to *streptococcal* stimulation and release CGRP, which inhibits neutrophil recruitment and leads to exacerbation of the local infection [11]. Interestingly, this negative regulation is present not only in infectious inflammation but also in noninfectious inflammation. In a rat model of arthritis, specific activation of TRPV1^+^ neurons by capsaicin injection inhibits M1 polarization in the synovial membrane via the Ca2 + /CaMKII/Nrf2 signaling pathway, thereby delaying the progression of osteoarthritis [34]. However, this neuromodulation is not always negative in regard to immunity. In the absence of risk factors for inflammation, activation of TRPV1^+^ neurons by optogenetic techniques alone can cause type 17 inflammation in innervated areas, and this inflammatory response is sufficient to defend against cutaneous *Candida albicans* and *S. aureus* infections [35]. The body’s immune response to invading microbes is homeostatically maintained [36], but the mechanism by which nociceptor neurons distinguish between different injurious stimuli and modulate the immune system to achieve this homeostasis needs to be further explored.

Previous in vitro experiments described that CGRP regulated macrophage polarization via the calmodulin, PKC and PKA pathways, and this regulation decreased LPS and adenosine triphosphate-induced secretion of NLRP3 and IL-1β from macrophages but increased IL-4-induced secretion of the macrophage M2 marker IL-10 [37]. This is similar to the results of the present study, and we further confirmed that the same regulation of macrophage polarization by CGRP exists in vivo. However, the regulation of skin immune cells by CGRP does not occur in macrophages alone, and Chiu et al. improved the outcome of skin infections in mice by blocking the action of CGRP in a model of *S. aureus* and *Streptococcus* skin infection, similar to the results of the present study, which suggested that this was caused by the inhibition of local recruitment of neutrophils by CGRP during infection [10, 11]. In the present study, we also observed differences in the amount of local neutrophil recruitment. In addition, we observed that CGRP inhibited the polarization of M1 macrophages and decreased TNF-α levels. The study by Gomes RN et al. found that the inhibition of neutrophil recruitment by CGRP may be indirect [38]. Neutrophils are produced and stored in the bone marrow and are ready to be transported through the circulatory system to the infected area upon injury [39]; however, recruited neutrophils tend to be short-lived, and are unable to remain as resident cells in the skin tissue for a long period of time [40]. In contrast, macrophages can reside in tissues for longer periods of time to monitor pathogen invasion through factors such as granulocyte–macrophage colony-stimulating factor and TNF-α [41]. Following microbial invasion, macrophages can be activated and produce neutrophil chemokines, and the recruited neutrophils can further release inflammatory factors that amplify the inflammatory response cascade [42]. Therefore, we speculate that skin macrophages act as “sentinels”, and may be regulated by TRPV1^+^ neurons before neutrophils during infection. Indeed, the synergistic action of neutrophils and macrophages enhances the body’s defense against pathogens, and the present study contributes to the understanding of the mechanism by which nociceptor neurons regulate macrophages in SSTI.

This study provides two potential drugs that could be used to treat SSTI: BoNT/A and BIBN4096. BoNT/A blocks neurotransmitter action by inhibiting vesicle release from neurons and has no effect on the excitability of sensory neurons. In the past decades, it has been used in the treatment of myasthenia gravis as well as facial plastic surgery and has been shown to have suitable efficacy and safety [43]. In recent years, BoNT/A has been used in wound healing, osteoarthritis and other conditions related to inflammation [44]. Intra-articular injection of BoNT/A in patients with knee osteoarthritis relieves pain and improves the function of the joint, which is thought to be due to the blockade of local CGRP and substance P release by BoNT/A [45]. Here, after we pretreated WT mice with BoNT/A, the outcome of SSTI was improved, suggesting BoNT/A as a potential therapeutic in the treatment of SSTI. BIBN4096 is an antagonist of CGRP that was developed for the treatment of migraine [46]. Previous studies found that CGRP antagonists increased local inflammation levels in skin scar tissue, leading to scar hyperplasia [47]. Similarly, when we used BIBN4096 to antagonize CGRP receptors to treat infected WT mice, the outcome of SSTI was improved. This indicates that BIBN4096 may be used in SSTI caused by *S. aureus*.

There are some limitations to this study. Firstly, we do not use the littermate control, which may lead to some unpredictable confounding factors. Secondly, some signaling molecules can have significant effects on the neuroimmune system, including TGF- β and some neurotransmitters (such as substance P), which have not been further explored in this study. In addition, we have not further explored the molecular pathway through which macrophages perceive CGRP, which needs to be further studied.

In conclusion, our study illustrates that TRPV1^+^ neurons inhibit neutrophil recruitment and modulate macrophage polarization by releasing CGRP in response to *S. aureus* infection, leading to exacerbation of local infection. How the sensory nervous system characterizes injurious stimuli and modulates the immune system through neural circuits are important questions that need to be further addressed.

## Data Availability

Data will be made available on request.
